# Texture Engineering Modulating Electromechanical Breakdown in Multilayer Ceramic Capacitors

**DOI:** 10.1002/advs.202300320

**Published:** 2023-04-07

**Authors:** Jian Wang, Zhong‐Hui Shen, Run‐Lin Liu, Yang Shen, Long‐Qing Chen, Han‐Xing Liu, Ce‐Wen Nan

**Affiliations:** ^1^ State Key Laboratory of Advanced Technology for Materials Synthesis and Processing Center of Smart Materials and Devices Wuhan University of Technology Wuhan 430070 China; ^2^ International School of Materials Science and Engineering Wuhan University of Technology Wuhan 430070 China; ^3^ School of Materials Science and Engineering State Key Lab of New Ceramics and Fine Processing Tsinghua University Beijing 100084 China; ^4^ Department of Materials Science and Engineering The Pennsylvania State University University Park Pennsylvania PA 16802 USA

**Keywords:** electromechanical breakdown, energy storage, machine learning, multilayer ceramic capacitors, texture engineering

## Abstract

Understanding the electromechanical breakdown mechanisms of polycrystalline ceramics is critical to texture engineering for high‐energy‐density dielectric ceramics. Here, an electromechanical breakdown model is developed to fundamentally understand the electrostrictive effect on the breakdown behavior of textured ceramics. Taking the Na_0.5_Bi_0.5_TiO_3_‐Sr_0.7_Bi_0.2_TiO_3_ ceramic as an example, it is found that the breakdown process significantly depends on the local electric/strain energy distributions in polycrystalline ceramics, and reasonable texture design could greatly alleviate electromechanical breakdown. Then, high‐throughput simulations are performed to establish the mapping relationship between the breakdown strength and different intrinsic/extrinsic variables. Finally, machine learning is conducted on the database from the high‐throughput simulations to obtain the mathematical expression for semi‐quantitatively predicting the breakdown strength, based on which some basic principles of texture design are proposed. The present work provides a computational understanding of the electromechanical breakdown behavior in textured ceramics and is expected to stimulate more theoretical and experimental efforts in designing textured ceramics with reliable electromechanical performances.

## Graphical abstract

A comprehensive electromechanical breakdown model is established to investigate the electrostrictive effect on the breakdown behavior of textured ceramics. Based on high‐throughput simulations, machine learning is performed to find the optimization strategy of texture engineering to guide the design of high energy storage performance ceramics.

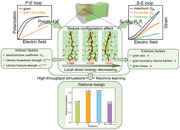

## 1 Introduction

Electrical energy storage devices, which store energy and release it on various demands, have become the essential components of modern electronics and electrical power systems.^[^ ^1^, ^2^, ^3^, ^4^, ^5^ ^]^ Compared to electrochemical supercapacitors, fuel cells, and rechargeable batteries, ceramic capacitors have the remarkable features of ultrahigh power density and excellent fatigue resistance, enabling broad applications ranging from high‐power microwaves to electromagnetic pulse weapons and hybrid electrical vehicles.^[^ ^6^, ^7^, ^8^, ^9^ ^]^ However, the relatively low energy density has greatly hindered the employment of higher energy storage requirements.

**Figure 1:**
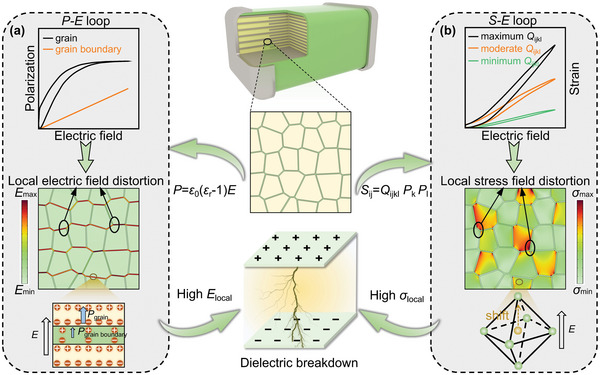
The schematic diagram of the electromechanical breakdown of dielectric ceramics based on a) the electrical response and b) the mechanical response.

The energy density of a dielectric capacitor is mainly determined by the electric‐field‐induced polarization and the breakdown strength,^[^ ^10^, ^11^, ^12^, ^13^, ^14^ ^]^ as the polarization–electric field loop (*P*–*E* loop) illustrated in **Figure** **1**a. However, the inverted relation between them has been a major obstacle to the increase in energy density. One of the overlooked but important reasons for not being able to raise both the polarization and the breakdown strength simultaneously is the coupling effect between electricity and force in ceramics.^[^ ^15^, ^16^ ^]^ As shown in Figure 1b, when applying an electric field on dielectric ceramics, the generation of polarization is accompanied by the output of strain by electrostrictive effect,^[^ ^17^ ^]^ which may trigger the electromechanical breakdown in brittle ceramics at a high electric field.^[^ ^15^, ^18^ ^]^ The electric‐field‐induced strain (*S*_ij_) of ceramics can be calculated by *S*_ij_ = *Q*_ijkl_ *P*_k_ *P*_l_, where *Q*_ijkl_ and *P*_i_ represent the electrostrictive coefficient and polarization, respectively. For example, if a dielectric ceramic with *Q*_3333_ up to 0.05 m^4^ C^−2^ is assembled into multilayer ceramic capacitors (MLCC) under an applied electric field of ≈50–80 MV m^−1^, the induced polarization exceeds 0.5 C m^−2^ and a giant average electrostrictive strain of more than 1% could be produced. More importantly, in polycrystalline ceramics, the large mismatches of electrical and mechanical features between grains and grain boundaries could further result in severe aggregation of the local electric/stress field, thus forming lots of weak points to induce the electromechanical breakdown. Therefore, reducing the electric‐field‐induced strain is helpful to avoid the premature occurrence of electromechanical breakdown and thus enhance the breakdown strength of MLCC.

Recently, to break the inversion relationship between the polarization and the breakdown strength, a lot of efficient methods have been successfully developed to increase the energy density, such as domain engineering,^[^ ^19^, ^20^, ^21^, ^22^ ^]^ high‐entropy strategy,^[^ ^23^, ^24^ ^]^ and composite structure design.^[^ ^25^, ^26^, ^27^, ^28^, ^29^ ^]^ However, most of them mainly focus on the influence of electric‐field‐induced polarization on the breakdown strength but ignore the influence of electric‐field‐induced strain. To this end, based on the strong electrostriction anisotropy in the perovskite structure,^[^ ^17^ ^]^ a texture engineering in Na_0.5_Bi_0.5_TiO_3_‐Sr_0.7_Bi_0.2_TiO_3_ (NBT‐SBT) multilayer ceramics was proposed to reduce the electric‐field‐induced strain and thus alleviate the electromechanical breakdown of MLCC.^[^ ^15^ ^]^ It was found that <111>‐textured NBT‐SBT MLCC exhibits the lowest *S*_33_ among all samples due to the minimum *Q*_3333_ along <111> direction. As a result, the breakdown strength of <111>‐textured ceramics was ≈65% higher than that of nontextured counterparts, and a record recoverable energy density of 21.5 J cm^−3^ was achieved at 103 MV m^−1^. Therefore, texture engineering has been proven to be a feasible and practical strategy to improve the breakdown strength and energy storage density of ceramics in MLCC by regulating the electrostriction response. However, an in‐depth understanding of how the texturing design affects the electromechanical breakdown process and energy storage performance of ceramics is still lacking.^[^ ^30^ ^]^ Therefore, figuring out the structure–property relationship in textured ceramics is highly desired to further guide subsequent experimental design.

In this work, we develop an electromechanical breakdown model to simulate the evolution of the breakdown path in textured ceramics by coupling the electrostrictive effect. Taking NBT‐SBT multilayer ceramics as an example, we first study the effect of texture configuration on the distributions of local electric/stress fields and corresponding energy density in ceramics. Then, we systematically simulate the electromechanical breakdown process by considering different intrinsic material parameters and extrinsic factors such as polycrystalline microstructures. Finally, machine learning is conducted to obtain an analytical expression to semi‐quantitatively evaluate the electromechanical breakdown behaviors in textured ceramics and conclude some basic principles of texture design. The present work not only reveals the underlying mechanism of electromechanical breakdown in textured ceramics but also establishes a theoretical rule of texture engineering to guide the design of dielectric ceramics with enhanced breakdown strength.

## 2 Results and Discussion

### 2.1 Texture Configuration Effect

**Figure 2:**
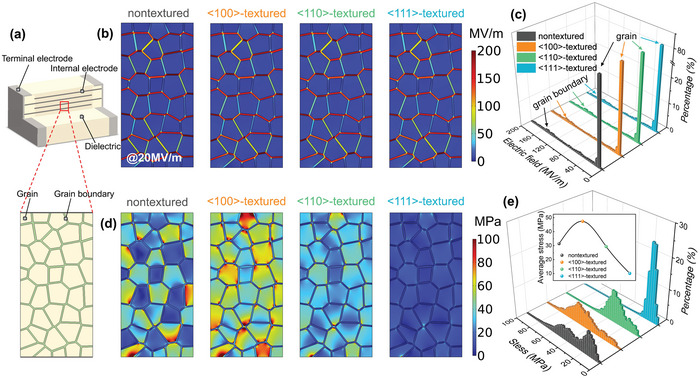
a) The schematic diagram and the selected typical region of MLCC. Local distributions of b) electric field and d) stress field of four samples under 20 MV m^−1^. The statistical distributions of c) local electric field and e) local stress field of four samples under 20 MV m^−1^.

First, we design four NBT‐SBT samples with different texture configurations to study the local electrical and mechanical responses, namely <100>‐textured, <110>‐textured, <111>‐textured, and nontextured ceramics. As shown in **Figure** **2**a, a rectangular region in MLCC with a grain (in yellow) and grain boundary (in green) is enlarged as the initial microstructure to simulate the local distributions of electric and stress fields. Figure 2b shows the electric field distributions of four ceramic samples under an applied electric field of 20 MV m^−1^. It can be seen that the dielectric mismatch between the grain and grain boundary results in local electric field distortion, and much higher electric fields are applied on the grain boundary. Nevertheless, the electric field distributions of the four samples with different grain orientations are almost identical, which is mainly attributed to the fact that the dielectric constant of different texture configurations varies very little. This is also evidenced by the statistical distributions of the local electric field in Figure 2c. The higher peaks located at the low electric field (≈8 MV m^−1^) correspond to the local electric field inside the grain, while the lower peaks located at the high electric field (≈87 MV m^−1^) represent the local electric field inside the grain boundary. For different texture configurations from <100> to <111>, the peak position, as well as the peak value, do not shift significantly. Therefore, the texture configurations have no significant effect on distorting the local electric field distribution in ceramics.

On the contrary, texture configuration greatly affects the local stress field distribution due to different *Q*_ijkl_ along different grain orientations, as shown in Figure 2d. In the nontextured ceramic sample, the high local stress field is mainly concentrated in the <100>‐oriented grains with higher *Q*_ijkl_. When all grains in ceramics orient from <100> to <111>, not only the stress field distribution becomes more homogeneous but also the average stress gets lower. This is because the fully <111>‐oriented grains with the lowest *Q*_ijkl_ significantly reduce the local mismatch of electrostrictive effect between different grains and the effective *Q*_ijkl_ of the whole sample. As plotted in Figure 2e, with the decrease of *Q*_ijkl_ from the nontextured sample to <111>‐textured sample, the peak of stress field distribution gradually changes from diffuse to sharp, which is an indicator of the enhanced homogeneity of the local stress field. In addition, the peak position also shifts toward lower values and the peak intensity becomes larger, demonstrating the reduction of the local stress field. Thus, the average stress of <111>‐textured sample (≈10 MPa) is much lower than that of <100>‐textured sample (≈47 MPa) at 20 MV m^−1^, as shown in the inset of Figure 2e. In addition, the effect of texture configuration on the average electric/stress field inside the grains and grain boundaries under the increased applied electric field is further investigated and displayed in Figure S2, Supporting Information. Similar to the above discussion, the texture configuration significantly affects the local stress field but not the local electric field. Therefore, the simulation results of local electrical and mechanical responses of four ceramic samples reveal that texture configuration could remarkably modify the local stress response with almost no change in the local electric response.

**Figure 3:**
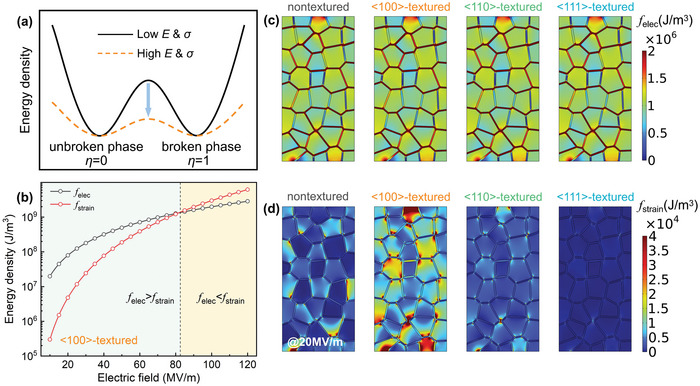
a) Variations of energy profile of unbroken phase and broken phase under physical stimuli. b) Comparisons of the maximum local electric energy density and the strain energy density at different applied electric fields in <100>‐textured sample. Local distributions of c) electric energy and d) stress energy of four samples under 20 MV m^−1^.

Based on the phenomenological theory of dielectric breakdown,^[^ ^31^ ^]^ the breakdown process can be analyzed from the double‐well energy profile, as presented in **Figure** **3**a. The two wells represent the unbroken phase (*η* = 0) and broken phase (*η* = 1), respectively, and the energy barrier between the unbroken phase and broken phase determines the difficulty of the occurrence of breakdown. As the external physical stimuli, such as the electric field *E* or stress *σ* or both, increase, the energy barrier decreases and the energy profile gradually flattens. When the physical stimuli are sufficiently large, the energy barrier between the unbroken phase and broken phase disappears and then the breakdown occurs. As the most important components of the total energy involved in dielectric breakdown, the competition between the electric and the strain energy densities can be used to understand the electromechanical breakdown process in ceramics. Taking <100>‐textured ceramic sample as the example, the maximum local electric and strain energy density as functions of the applied electric field is shown in Figure 3b. All energy densities increase with the electric field, and the increased rate of strain energy is faster than that of electric energy. At low electric fields, the electric energy is larger than the strain energy, suggesting that the electrostatic effect dominates the breakdown process. When the applied electric field is larger than 81 MV m^−1^, the strain energy exceeds the electric energy, thus the breakdown mechanism changes from electrostatic breakdown to electromechanical breakdown due to the electrostrictive effect. This change in the breakdown mechanism could also be reflected in the maximum local energy density, as shown in Figure S3, Supporting Information. The increase of the strain energy density enhances the probability of crack‐driven dielectric breakdown, leading to the deterioration in the electromechanical breakdown strength. Therefore, the breakdown process is closely associated with the local electric and strain energy density.

Then, the distributions of local electric energy density (*f*_elec_) and strain energy density (*f*_strain_) for four ceramic samples with different texture configurations under 20 MV m^−1^ are also quantitatively displayed in Figure 3c,d. For the electric energy density shown in Figure 3c, similar to the above simulated electric field distributions in Figure 2b, it is less dependent on the texture configuration because the dielectric constant of grain changes only slightly from <100> to <111>. For instance, the average electric energy density of <100>‐textured ceramic sample is ≈1.49 × 10^6^ J m^−3^, which has a similar value to ≈1.45 × 10^6^ J m^−3^ of <111>‐textured ceramic sample. However, the strain energy densities under different texture configurations are completely different, as shown in Figure 3d. Due to the high dependence of *Q*_ijkl_ on grain orientation, *Q*_1111_ and *Q*_1122_ of the textured ceramics are reduced from 0.05 and −0.02 m^4^ C^−2^ of the <100> direction to 0.015 and −0.004 m^4^ C^−2^ of the <111> direction, respectively. Therefore, the strain energy density distribution of <111>‐textured sample is much lower and more homogeneous than that of other samples, and the average strain energy density decreased sharply from 1.61 × 10^4^ to 690 J m^−3^ as the textured direction changes from <100> to <111>. Thus, regulating the texture structure can lead to a lower and more homogeneous strain energy distribution, which is more conducive to delaying and suppressing the premature occurrence of electromechanical breakdown.

### 2.2 Electromechanical Breakdown Process

**Figure 4:**
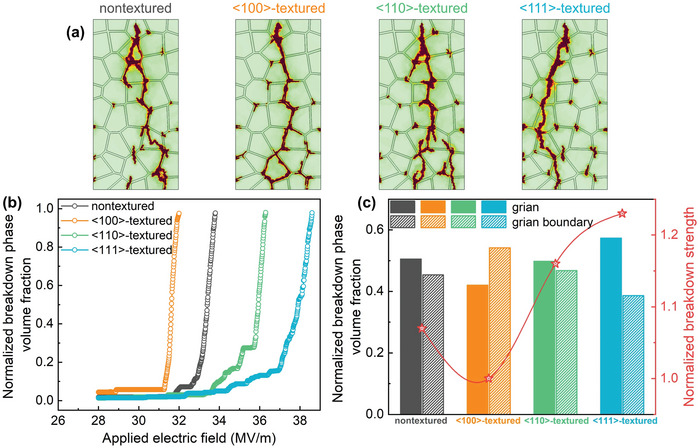
a) The evolution of electrical trees of four samples. b) Variations of normalized breakdown phase volume fraction of four samples under applied electric field. c) Comparisons of the normalized breakdown phase volume fraction distribution and the normalized breakdown strength of four samples.

The above simulation results indicate that the texture configuration can significantly affect the local stress field and corresponding strain energy density in ceramics. Here, in order to further analyze the electromechanical breakdown process, we perform a 2D simulation to predict the breakdown phase evolution under applied electric fields, and the electrical trees of four ceramic samples at the final broken state are shown in **Figure** **4**a. It is found that the differences in local energy density distribution among different textured ceramics result in totally different breakdown behaviors. For example, as <100>‐textured sample with the high local strain energy density displayed in Figure 3d, the strain energy is predominant and grain boundaries are prone to become hot spots during the breakdown process, resulting in faster propagation of breakdown phase and eventually a complete breakdown path along the grain boundaries is observed. This electromechanical breakdown behavior is similar to many experimental observations of brittle fracture in polycrystalline ceramics under tensile loading.^[^ ^32^, ^33^, ^34^ ^]^ Nevertheless, the breakdown path in <111>‐textured sample looks very different. The <111>‐textured sample exhibits lower and more homogeneous strain energy distributions among all ceramic samples under the same electric field, in this case, the electric energy is predominant. Thus, the propagation rate of the breakdown phase becomes slower without the contribution of the strain energy density, and the breakdown path prefers to penetrate through the grains since the grains have worse endurance capability of electric field than the grain boundaries. Therefore, as the local strain energy density in textured ceramics decreases, completely different breakdown behaviors are observed. The breakdown path of strain‐energy‐dominated electromechanical breakdown in <100>‐textured sample is more distributed in the grain boundaries, the ceramic shows the characteristics of intergranular breakdown. While for the electric‐energy‐dominated electrostatic breakdown process of <111>‐textured sample, the breakdown path tends to appear in the grains, and the breakdown mode transforms from intergranular breakdown to transgranular breakdown.

Then, we calculate the volume fraction of the breakdown phase under increasing electric fields to quantitatively evaluate the breakdown process, as displayed in Figure 4b. The first inflection point on the curve indicates the electric field where the breakdown phase begins to grow, then the breakdown phase grows rapidly as the electric field increases until complete breakdown. The simulated evolution of the normalized breakdown phase shows different growth rates versus texture configuration, depending on the breakdown mechanisms that affected by local energy density. For <100>‐textured sample, due to the high local strain energy distributions, the electrical‐mechanical coupling effect appears in the breakdown process. Thus, <100>‐textured ceramic sample shows the fastest growth rate of the breakdown phase, and the normalized breakdown phase volume fraction reaches a maximum when the electric field is ≈31.5 MV m^−1^. In contrast, with the benefit from the lower and homogeneous local strain energy distributions, the electrostatic effect is predominant in the breakdown process of <111>‐textured ceramic sample. So <111>‐textured ceramic sample has the slowest growth rate of breakdown phase driven by only electrostatic energy and not be broken totally until the applied electric field reaches ≈38.7 MV m^−1^, implying that <111>‐textured ceramic sample has the largest breakdown strength in comparison to other counterparts. Then, the breakdown phase distribution is also illustrated in Figure 4c to further describe the difference in the breakdown behaviors in textured ceramics. Generally, the grain boundaries are considered amorphous regions that are more disordered and defective than the crystalline grains,^[^ ^33^, ^35^ ^]^ and therefore, the intrinsic fracture strength $\sigma_{b}^{\mathrm{intrinsic}}$ of grain boundary is lower than that of grain. As the local strain energy distributions of <100>‐textured ceramic sample shown in Figure 3d, the severe accumulation of the local strain energy at grain boundaries makes those regions become hot spots easily and then promotes the rapid propagation of the breakdown path along the grain boundaries. As a result, the breakdown path of <100>‐textured ceramic sample is mainly distributed in the grain boundaries. When all grains orient to <111>, <111>‐textured ceramics with the lowest *Q*_ijkl_ present clearly lower local strain energy and reduce the possibility of cracks at grain boundaries. Afterward, the breakdown path prefers to propagate across the grains because the intrinsic breakdown strength $E_{b}^{\mathrm{intrinsic}}$ of the grain is smaller than that of the grain boundary.^[^ ^36^, ^37^, ^38^ ^]^ Thus, the evolution of the breakdown phase can be effectively suppressed by reducing the strain energy, resulting in a higher breakdown strength of <111>‐textured sample than that of <100>‐textured sample. This discovery is consistent with recent experimental results, in which Li et al. fabricated high‐quality <111>‐textured NBT‐SBT ceramics by grain orientation engineering.^[^ ^15^ ^]^ It was found that the <111>‐textured sample showed a greatly lowered electric‐field‐induced strain, which reduces the probabilities of the initiation of microcracks and delays the growth speed of the breakdown path. Consequently, the <111>‐textured NBT‐SBT multilayer ceramic exhibited greatly enhanced breakdown strength and record‐high recoverable energy density compared to other textured counterparts. Therefore, reducing the electric‐field‐induced strain means that the electrical reliability and fatigue endurance of dielectric ceramics are improved, and texture engineering makes full use of this advantage to increase the breakdown strength to a higher value.

### 2.3 Intrinsic Factors in Texture Engineering

As discussed above, the electromechanical failure process of textured ceramics depends on material parameters such as electrostrictive coefficient *Q*_ijkl_, intrinsic breakdown strength $E_{b}^{\mathrm{intrinsic}}$, and intrinsic fracture strength $\sigma_{b}^{\mathrm{intrinsic}}$. Lower *Q*_ijkl_ could lead to a smaller electrostrictive strain and relieve the aggregation of the local stress field to inhibit the emergence of microcracks. High $E_{b}^{\mathrm{intrinsic}}$ makes ceramics withstand higher applied electric field which is quadratically correlated to the recoverable energy density. High $\sigma_{b}^{\mathrm{intrinsic}}$ could resist large local electric‐field‐induced strain and greatly decrease the electromechanical breakdown probability of ceramics. Thus, the electromechanical breakdown behavior can be altered by controlling these intrinsic factors, and it is necessary to explore the effect of those intrinsic factors on the breakdown process to guide experimental design.

**Figure 5:**
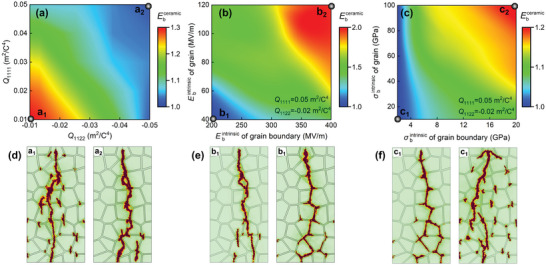
High‐throughput simulations of $E_{b}^{\mathrm{creamic}}$: dependences of $E_{b}^{\mathrm{creamic}}$ on a) *Q*_1111_ and *Q*_1122_, b) $E_{b}^{\mathrm{intrinsic}}$ of grain and grain boundary, and c) $\sigma_{b}^{\mathrm{intrinsic}}$ of grain and grain boundary. The evolution of electrical trees of d) point a1 and a2 in (a), e) point b1 and b2 in (b), and f) point c1 and c2 in (c).

Here, by performing high‐throughput simulations, the effects of *Q*_ijkl_, $E_{b}^{\mathrm{intrinsic}}$, and $\sigma_{b}^{\mathrm{intrinsic}}$ on the breakdown strength are systematically investigated and mapped in **Figure** **5**. From the map in Figure 5a, it can be seen that the increase of *Q*_ijkl_ is detrimental to the breakdown strength of textured ceramic $E_{b}^{\mathrm{creamic}}$, as $E_{b}^{\mathrm{creamic}}$ gradually goes down from the left bottom to the right upper of the mapping. This can be understood from the local distributions of the stress field and corresponding strain energy density under different *Q*_ijkl_ shown in Figure S4, Supporting Information. The increase of *Q*_ijkl_ leads to more severe local stress field/strain energy distortion in textured ceramics, and thus decreases the breakdown strength. Taking points a1 and a2 as examples in Figure 5d, as *Q*_ijkl_ increases, the contribution of strain energy enhances and becomes more and more important, and the growth of the breakdown path gradually changes from along the grains to along the grain boundaries. With *Q*_1111_ and *Q*_1122_ increasing from 0.01 and −0.01 m^4^ C^−2^ to 0.05 and −0.05 m^4^ C^−2^, the normalized $E_{b}^{\mathrm{creamic}}$ decreases from 1.3 to 1.0. Therefore, $E_{b}^{\mathrm{creamic}}$ is highly negatively correlated with *Q*_ijkl_. Next, we take <100>‐textured ceramic as the example to investigate how $E_{b}^{\mathrm{intrinsic}}$ and $\sigma_{b}^{\mathrm{intrinsic}}$ of grain and grain boundary affect the breakdown process. As shown in Figure 5b, $E_{b}^{\mathrm{creamic}}$ increases by nearly two times when $E_{b}^{\mathrm{intrinsic}}$ of grain and grain boundary changes from 40 and 200 MV m^−1^ to 120 and 400 MV m^−1^, indicating that higher $E_{b}^{\mathrm{intrinsic}}$ gives rise to higher $E_{b}^{\mathrm{creamic}}$. Moreover, it is found that $E_{b}^{\mathrm{intrinsic}}$ also has a strong effect on the growth behavior of the breakdown path, and the breakdown paths corresponding to points b1 and b2 are displayed in Figure 5e. When $E_{b}^{\mathrm{intrinsic}}$ is low, the voltage endurance of the textured ceramics decreases and dielectric failure is more likely to occur at low electric fields. Although *Q*_ijkl_ is large, the electrostrictive strain which is quadratically correlated to the electric field is still not significant under low electric voltage, thereby the breakdown path mainly expands along the grains. When $E_{b}^{\mathrm{intrinsic}}$ becomes higher, the electric field applied on the textured ceramics becomes larger, the electrostrictive effect is intensified and the local strain energy is distorted, thus the breakdown phase mainly expands along the grain boundaries. Therefore, for ceramics with high $E_{b}^{\mathrm{intrinsic}}$, the strain and strain energy induced by the applied electric field cannot be neglected. The effect of $\sigma_{b}^{\mathrm{intrinsic}}$ on the breakdown behavior is also studied and displayed in Figure 5c. The mapping results show a clear positive correlation between $E_{b}^{\mathrm{creamic}}$ and $\sigma_{b}^{\mathrm{intrinsic}}$. When $\sigma_{b}^{\mathrm{intrinsic}}$ of grain and grain boundary approaches its Young's modulus, $E_{b}^{\mathrm{creamic}}$ can be increased by ≈1.2 times. Additionally, the breakdown path also changes with $\sigma_{b}^{\mathrm{intrinsic}}$. An increase in $\sigma_{b}^{\mathrm{intrinsic}}$ could improve the ability to resist the giant electrostrictive strain under repetitive electric voltage loading, which is beneficial to inhibit the initiation of cracks at grain boundaries and enhance $E_{b}^{\mathrm{creamic}}$. Thus, the growth of the breakdown path gradually varies from along the grain boundaries to along the grains as $\sigma_{b}^{\mathrm{intrinsic}}$ increasing, as shown by the breakdown path corresponding to points c1 and c2 in Figure 5f.

Therefore, $E_{b}^{\mathrm{creamic}}$ can be improved by changing the intrinsic factors such as lowering *Q*_ijkl_ and raising $E_{b}^{\mathrm{intrinsic}}$ and $\sigma_{b}^{\mathrm{intrinsic}}$. In experiments, many strategies have been proposed to achieve these performance targets. For example, the template grain growth method can be adopted to fabricate textured ceramics with the lowest *Q*_ijkl_ by controlling grain orientation,^[^ ^15^, ^30^ ^]^ leading to a reduced electrostrictive strain and improved breakdown strength. The formation of a solid solution by introducing wide band gap materials, such as HfO_2_ and Ta_2_O_5_, to prevent electrons jumping from the top of the valence band to the bottom of the valence band, thereby increasing $E_{b}^{\mathrm{intrinsic}}$.^[^ ^23^, ^39^, ^40^, ^41^ ^]^ The addition of numerous ions with different ionic radii can design heterostructures to enhance the local random stress field to effectively impede the motion of dislocations, which results in an enhanced macroscopic $\sigma_{b}^{\mathrm{intrinsic}}$.^[^ ^19^, ^42^ ^]^

### 2.4 Extrinsic Factors in Texture Engineering

Dielectric breakdown, as a high nonequilibrium complex process, depends not only on the intrinsic factors of ceramics but also on extrinsic factors, such as defects, as well as variations in morphology and microstructure. At the microscopic scale, it has been revealed that the overall measured properties of polycrystalline ceramics are closely related to its microstructure, including grain size, grain boundary, and grain shape.^[^ ^16^, ^36^, ^43^, ^44^, ^45^, ^46^, ^47^, ^48^ ^]^ Clearly, systematically evaluating these microstructure effects would help to understand the electrical and mechanical properties of textured ceramics and provide guidance for further optimization of microstructure to improve the breakdown strength.

**Figure 6:**
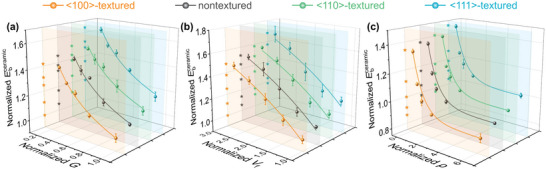
Normalized $E_{b}^{\mathrm{creamic}}$ of four samples as a function of a) normalized *G*, b) normalized *V*_f_, and c) normalized *ρ*.

In the following, we investigate the microstructure effects of grain size, grain boundary, and grain shape on the breakdown strength of textured ceramics. Although many experimental literatures have reported that grain size has an important impact on the dielectric properties of ceramics,^[^ ^36^, ^45^, ^49^ ^]^ the analysis of size‐dependent electromechanical breakdown mechanism is still lacking. Here, five ceramic models with different grain sizes (*G*) are illustrated in Figure S5a, Supporting Information, and the relationship between $E_{b}^{\mathrm{creamic}}$ and *G* is simulated and shown in **Figure** **6**a. It can be easily found that with the decrease of *G*, $E_{b}^{\mathrm{creamic}}$ with different texture configurations will be significantly enhanced. Taking <100>‐textured ceramic as an instance, the normalized $E_{b}^{\mathrm{creamic}}$ with a normalized *G* of 0.32 is almost 42% higher than that with a normalized *G* of 1. The local electric/stress field distributions are shown in Figure S5b,c, Supporting Information, which can be used to understand this finding. Under a given applied electric field, the distribution of the local field is very sensitive to the grain size. As *G* decreases, the local electric field is homogenized and lowered. Then the local stress field is reduced and the average stress decreases from ≈47 to ≈12 MPa as the normalized *G* varies from 1 to 0.32, which subsequently leads to the change of the breakdown path, as shown in Figure S8a, Supporting Information. Therefore, a more homogeneous local electric/stress field distribution caused by smaller *G* will lead to higher $E_{b}^{\mathrm{creamic}}$. Grain boundary, as another extrinsic factor, is helpful to enhance the breakdown strength because it favors higher electrical resistivity.^[^ ^38^, ^50^, ^51^ ^]^ Five models with different grain boundary volume fractions (*V*_f_) are also considered and displayed in Figure S6, Supporting Information. When the normalized *V*_f_ increases to 2.76, the inhomogeneities of both local electric and stress fields are weakened, especially the average stress decreases by approximately five times, which can be used to explain the breakdown behaviors under different normalized *V*_f_ in Figure S8b, Supporting Information. Therefore, with the normalized *V*_f_ increasing from 1 to 2.76, the normalized $E_{b}^{\mathrm{creamic}}$ of <100>‐textured ceramic sample is raised from 1 to 1.48, as shown in Figure 6b. Next, we turn to investigate the effect of grain shape on the breakdown behaviors. By controlling the shape of the initial nuclei, five polycrystalline structures with different aspect ratios are generated and plotted in Figure S7a, Supporting Information. We introduce a physics‐based descriptor, the shape factor (*ρ*), to describe the shape of the grain. As *ρ* varies from 0.1 to 10, the grain shape transforms from lateral to longitudinal. It is found that the effect of *ρ* on electromechanical response originates from the inhomogeneous electric field and stress induced by the external electric field, as shown in Figure S7b,c, Supporting Information. As *ρ* decreases, the distribution range of the local electric field decreases significantly. This is due to the increase in the area of lateral grain boundaries. The depolarization effect in flat grains which possess more area of lateral grain boundaries should be much weaker than that in regular grains, most electric voltage in the grain boundaries will be transferred to the grains, and then the local electric field becomes more homogeneous. The increased homogeneity of the electric field would decrease the magnitude of the stress field, which helps to mitigate electromechanical breakdown. Thus, the normalized $E_{b}^{\mathrm{creamic}}$ of <100>‐textured sample increases from 0.86 to 1.34 when the normalized *ρ* decreases from 6.29 to 0.29, as shown in Figure 6c.

Therefore, the above theoretical simulations reveal that the breakdown strength is highly dependent on the microstructures of textured ceramics. Designing polycrystalline microstructures with smaller *G*, higher *V*_f_, and lower *ρ* could improve $E_{b}^{\mathrm{creamic}}$ more effectively. Up to now, some advanced and efficient preparation techniques have shown great potential in the modification of the ceramic microstructure. For instance, various methods, such as sol–gel method and spark plasma sintering, have been employed to prepare dense ceramics with fine grains and uniform microstructure.^[^ ^52^, ^53^, ^54^, ^55^ ^]^ For grain boundary, chemical doping or coating highly insulating linear dielectrics, such as SiO_2_, Al_2_O_3_, and MgO, on the ceramic surface to form a heterogeneous core–shell microstructures could achieve the regulation of the grain boundary volume fraction.^[^ ^48^, ^51^, ^53^, ^56^ ^]^ With respect to grain shape, some experimental approaches, such as tuning sintering temperature and adding surfactant, have been proposed to form ceramics with tailored architectures.^[^ ^44^, ^57^ ^]^ Therefore, choosing suitable technologies to modulate the polycrystalline microstructure in ceramics is also a powerful method to improve the breakdown strength and energy density.

### 2.5 Machine Learning of Texture Engineering

**Figure 7:**
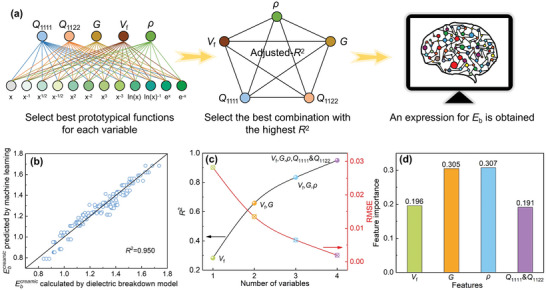
a) Schematic workflow of the machine learning for producing an analytical expression for $E_{b}^{\mathrm{creamic}}$. b) Comparisons of normalized $E_{b}^{\mathrm{creamic}}$ between the dielectric breakdown model and the machine learning prediction. c) Effects of independent variables on *R* ^2^ and RMSE in the regression model. d) Feature importance based on the weight in XGBoost.

To further clarify the effects of various factors in texture engineering, taking 180 groups of high‐throughput simulations as the dataset, we perform machine learning to find the analytical expression for $E_{b}^{\mathrm{creamic}}$ as a function of electrostrictive coefficient (*Q*_1111_ and *Q*_1122_), grain size (*G*), grain boundary volume fraction (*V*_f_), and shape factor (*ρ*). The workflow of the machine learning process for $E_{b}^{\mathrm{creamic}}$ is shown in **Figure** **7**a, 12 prototypical functions and 26 interactions are considered to generate descriptors to perform the regression analysis by least squares regression (LSR). Here, the coefficient of determination (*R* ^2^) of the LSR is used as the criterion for screening descriptors, and all variables are normalized to eliminate numerical differences to ensure that the obtained expression is applicable to a wider range of ceramics. After the fifth round of screening of LSR results, a simple and practical expression without considering the interaction among variables is obtained. The expression is given by

(1) $$\begin{array}{ccc} E_{b}^{ceramic} & = & 1.179-1.098Q_{1111}^{1/2}+0.392Q_{1122}+0.508G^{-1/2}\\ & & +\;0.068V_{f}^{2}-0.138\ln\rho \end{array}$$

This expression boasts a high coefficient of determination *R* ^2^ = 0.950 (see Tables S1–S5, Supporting Information), indicating that the accuracy of this machine‐learning model is very high. More details of the regression analysis can be found in the Supporting Information. This expression implies that $E_{b}^{\mathrm{creamic}}$ increases with *V*_f_ but decreases with *Q*_ijkl_, *G*, and *ρ*. Then, in order to test the reliability of the analytical expression for $E_{b}^{\mathrm{creamic}}$ generated from machine learning, Figure 7b plots the comparisons of $E_{b}^{\mathrm{creamic}}$ calculated by the dielectric breakdown model and $E_{b}^{\mathrm{creamic}}$ predicted by the machine learning model. It is found that most of the data points (marked as blue symbols) are dispersed around the black solid line, indicating that the predicted results are in good agreement with the simulated results. Therefore, Equation (1) can be employed to semi‐quantitatively predict the intrinsic and extrinsic factors' effects on $E_{b}^{\mathrm{creamic}}$.

Then, we are dedicated to discovering more important variables to effectively guide the experimental design of textured ceramics with higher breakdown strength, thus shortening the time and reducing the cost of the development of new dielectric materials. In general, *Q*_1111_ and *Q*_1122_ are highly positively correlated. We have found that *Q*_1111_ and *Q*_1122_ in dielectric ceramics vary simultaneously and the value of *Q*_1111_ is usually much larger than that of *Q*_1122_, as shown in Figure S9, Supporting Information. Therefore, we consider the correlation between *Q*_1111_ and *Q*_1122_ and combine them into a compound variable *Q*_1111_&*Q*_1122_. We first build a regression model from the dataset, and the performance is evaluated by calculating the root mean square error (RMSE) and the coefficient of determination (*R* ^2^). As shown in Figure 7c, when all variables are taken into account, *R* ^2^ and RSME with 0.951 and 0.0019, respectively, indicating that *Q*_1111_&*Q*_1122_, *G*, *V*_f_, and *ρ* are also the four variables that can together conspire to affect $E_{b}^{\mathrm{creamic}}$. Next, to determine the most relevant variables to $E_{b}^{\mathrm{creamic}}$, the weight‐type algorithm in eXtreme Gradient Boosting (XGBoost) regression is utilized to evaluate the effect of each feature on the performance of the model.^[^ ^58^, ^59^ ^]^ The importance of each feature is obtained and displayed in Figure 7d, it can be seen that variables *G* and *ρ* possess the high feature importance of 0.305 and 0.307, respectively, which are higher than that of variables *Q*_1111_&*Q*_1122_ and *V*_f_. The higher the feature importance value, the more important that feature is. Therefore, the different feature importance of the four variables indicates that *G* and *ρ* have more significant effects on $E_{b}^{\mathrm{creamic}}$. Based on the machine learning prediction, it can be concluded that $E_{b}^{\mathrm{creamic}}$ can be enhanced by designing polycrystalline microstructures with higher *V*_f_ but lower *G*, *ρ*, and *Q*_ijkl_. Moreover, under the premise of textured ceramics with lower *Q*_ijkl_ and higher *V*_f_, further reduction in *G* and *ρ* will be more critical to improve $E_{b}^{\mathrm{creamic}}$.

## 3 Conclusion

In summary, an electromechanical breakdown model is established to understand the electromechanical breakdown process arising from the electrostrictive effect in textured ceramics. It is found that the texture configuration would significantly affect the stress field and corresponding strain energy density distributions in textured ceramics, which eventually leads to completely different breakdown behaviors. The underlying mechanism of electromechanical breakdown is revealed, and the effects of intrinsic and extrinsic factors on the electrical and mechanical properties of textured ceramics are systematically studied. Then machine learning is performed to produce an analytical expression for $E_{b}^{\mathrm{creamic}}$ as a function of *Q*_ijkl_, *G*, *V*_f_, and *ρ*. This expression can be used to semi‐quantitatively predict the breakdown strength of textured ceramics with known material parameters and microstructure features. Furthermore, some basic principles of texture design have been proposed to help experimentally design high‐energy storage performance ceramics more efficiently. This work provides fundamental insights into the mechanism of electromechanical breakdown and is expected to stimulate more future efforts on synthesizing textured ceramics with excellent dielectric properties.

## 4 Experimental Section

### Dielectric Breakdown Model

In this work, a polycrystalline ceramic model, including grain (in yellow) and grain boundary (in green), was constructed, and the loading setup for the simulated model is shown in Figure S1, Supporting Information. When an electric field was applied to the ceramic, the local electric field distribution in the ceramic can be obtained by calculating the electrostatic Poisson's equation

(2) $$\nabla (\varepsilon_{0}\varepsilon_{r}E)=0$$

(3) E=−∇V

where *E*, *V*, *ɛ*_0_, and *ɛ*_r_ represent the local electric field, electric potential, vacuum dielectric constant, and relative dielectric constant, respectively. According to the experimental results,^[^ ^15^ ^]^ the dielectric constant of grain for <100>‐textured, <110>‐textured, and <111>‐textured were set to 2142, 2223, and 2303, respectively, while the dielectric constant of grain boundary was regarded as the linear dielectric with the value of 100. Neumann boundary conditions were applied on both sides of the model as

(4) $$\frac{\partial V}{\partial n}=0$$

where *n* is the unit normal vector. Based on the calculated local electric field, the electrostrictive effect was coupled into the stress equilibrium equation. Then the local stress field distribution was given by

(5) $$\rho \frac{\partial^{2}u}{\partial t}=\nabla \sigma +F_{V}$$

(6) σ=Yε

(7) $$\varepsilon =QP^{2}$$

where *ρ*, *u*, *σ*, *F*_V_, *Y*, *ɛ*, *Q*, and *P* represent the density, displacement vector, stress, volume force, Young's modulus, strain, electrostrictive coefficient, and polarization, respectively. Generally, grain boundaries were thought to be more defective regions and thus softer than grains, so Young's modulus of grain boundaries was smaller than that of grains. Here, Young's modulus of grains and grain boundaries were set as 100 and 20 GPa. In addition, only the electrostrictive effect of the grain was considered, the *Q*_1111_ of the <100>, <110>, and <111>‐oriented grain were set to 0.05, 0.03, and 0.015 m^4^ C^−2^, respectively, and the *Q*_1122_ of the <100>, <110>, and <111>‐oriented grain were set to −0.02, −0.012, and −0.004 m^4^ C^−2^, respectively.^[^ ^15^ ^]^

Then, to investigate the electromechanical breakdown behaviors in textured ceramics, a continuous variable *η*(r,t) was introduced to spatially and temporally describe the breakdown process in ceramics. The value of *η*(r,t) changed continuously from 0 to 1, where *η*(r,t) = 0 represents the unbroken phase and *η*(r,t) = 1 represents the broken phase. Thus, the dielectric breakdown can be described by defining the evolution of the normalized variable *η*(r,t) (0 ≤ *η*(r,t) ≤ 1) in the rate equation

(8) $$\frac{\partial \eta (r,t)}{\partial t}=A\eta^{2}-B\eta +C,\left(A,B,C\geq 0\right)$$

where *A*, *B*, and *C* decide the weight of each term in the equation. Theoretically, the electromechanical failure of dielectric ceramics was highly associated with local electric/strain energy density. Breakdown spots were easily triggered from regions of severe accumulation of the local electric/strain energy, while they were suppressed in regions of low electric/strain energy. Therefore, to describe the contribution of different energy densities, the model was rewritten by redefining the coefficient *C* as follows

(9) $$C=a\frac{f_{\mathrm{elec}}}{f_{\mathrm{elec}}^{\mathrm{critical}}}+b\frac{f_{\mathrm{strain}}}{f_{\mathrm{strain}}^{\mathrm{critical}}}$$

where $f_{\mathrm{elec}}^{\mathrm{critical}}$ and $f_{\mathrm{strain}}^{\mathrm{critical}}$ are position‐dependent material constants representing the critical electric energy density and critical strain energy density of the grain and grain boundary in polycrystalline ceramics, and *a* and *b* determine the contribution of electric and strain energy to the breakdown process. Here, it was assumed that dielectric breakdown occurred only when the energy density of the local point was greater than its critical energy, and the critical electric energy and critical strain energy of grain and grain boundary were calculated by $\varepsilon_{0}\varepsilon_{r}{\left(E_{b}^{\mathrm{intrinsic}}\right)}^{2}/2$ and ${\left(\sigma_{b}^{\mathrm{intrinsic}}\right)}^{2}/2Y$. Due to the lack of experimental data on $E_{b}^{\mathrm{intrinsic}}$ of grain and grain boundary, the rational values of 120 and 400 MV m^−1^ were assigned for grain and grain boundary. Theoretically, $\sigma_{b}^{\mathrm{intrinsic}}$ was related to Young's modulus of the ceramic, and $\sigma_{b}^{\mathrm{intrinsic}}$ can be written as $\sigma_{b}^{\mathrm{intrinsic}}={\left(Y\gamma /d\right)}^{1/2}$,^[^ ^60^ ^]^ where *γ* is the surface energy per unit area, and *d* is the atomic spacing. For ceramics, $\sigma_{b}^{\mathrm{intrinsic}}$ is estimated to be about *E*/5≈*Y*/10. Here, $\sigma_{b}^{\mathrm{intrinsic}}$ of grain and grain boundary were set to 10 and 2 GPa, respectively.

### Machine Learning Approach

Here, five factors, including electrostrictive coefficient (*Q*_1111_ and *Q*_1122_), grain size (*G*), grain boundary volume fraction (*V*_f_), and shape factor (*ρ*) were selected as independent variables to determine a cause‐effect relationship with the breakdown strength of textured ceramics ($E_{b}^{\mathrm{creamic}}$). As shown in Figure 7a, 12 prototypical functions and 26 interactions among variables were considered to generate candidate expressions and descriptors to conduct the regression analysis by LSR. Then, the coefficient of determination (*R* ^2^) was used as the criterion for ranking and screening the different expression candidates and descriptors, in order to obtain an analytical expression with the highest *R* ^2^.

To discover the most relevant variables to the breakdown strength, an eXtreme Gradient Boosting (XGBoost) algorithm was used to conduct a feature importance analysis based on four effective variables that can sufficiently describe the breakdown strength of textured ceramics. With the help of the common feature importance measurement parameters, that is, weight, the importance score for the input feature based on the weight‐type algorithm in XGBoost regression can be obtained.

## Conflict of Interest

The authors declare no conflict of interest.

## Supporting information

Supporting Information

## Data Availability

The data that support the findings of this study are available from the corresponding author upon reasonable request.
