# Supplementary material for: Texture Engineering Modulating Electromechanical Breakdown in Multilayer Ceramic Capacitors
Source: Adv Sci (Weinh). 2023 Apr 7;10(16):2300320. doi: 10.1002/advs.202300320 (PMC10238190; doi:10.1002/advs.202300320)
Supplement: Supplementary file 1 — Supporting Information [file ADVS-10-2300320-s001.pdf]

Supporting Information

Texture engineering modulating electromechanical breakdown in multilayer ceramic capacitors

Jian Wang, Zhong-Hui Shen, Run-Lin Liu, Yang Shen, Long-Qing Chen, Han-Xing Liu*, Ce-Wen Nan**

1. Schematics diagram of the modeling approach to simulate electromechanical breakdown processes in textured ceramics

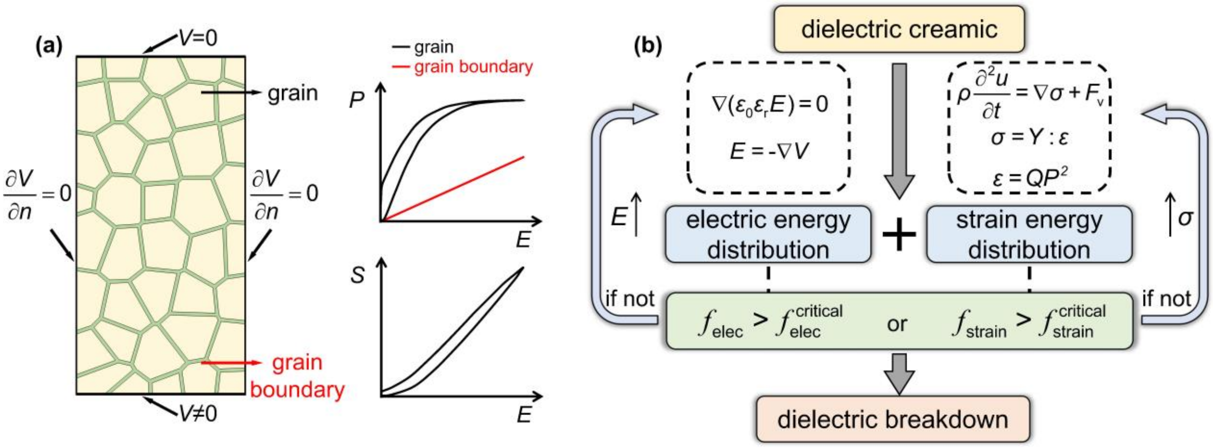

Figure S1. Schematic diagram of a) the loading setup and b) the breakdown process of ceramics in the dielectric breakdown model.

2. Effects of texture configuration on the average electric/stress field in grain and grain boundary

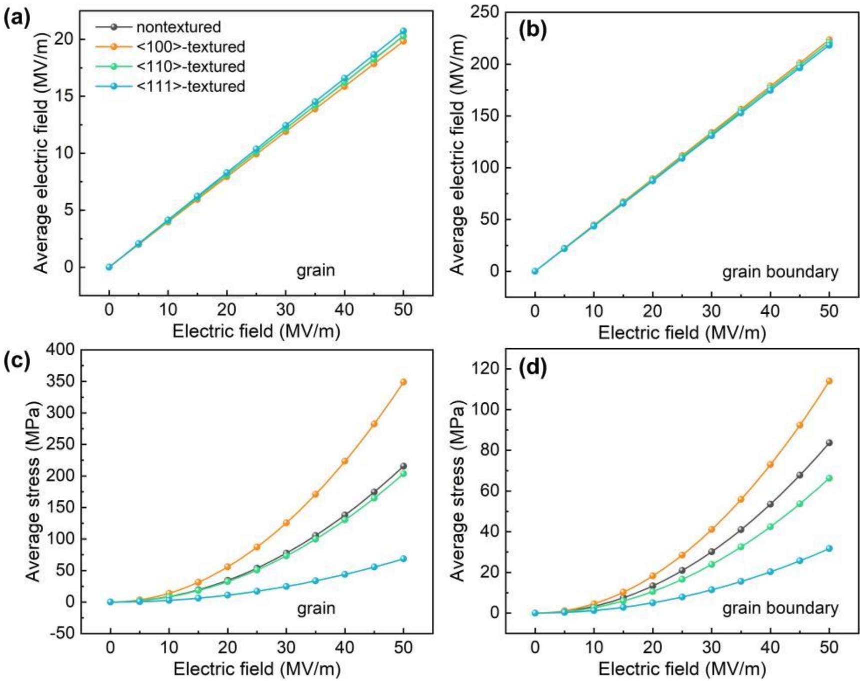

Figure S2. Comparisons of the average electric field in a) grain and b) grain boundary of four samples at different applied electric fields. Comparisons of the average stress in a) grain and b) grain boundary of four samples at different applied electric fields.

3. Effects of texture configuration on the energy density at different applied electric fields

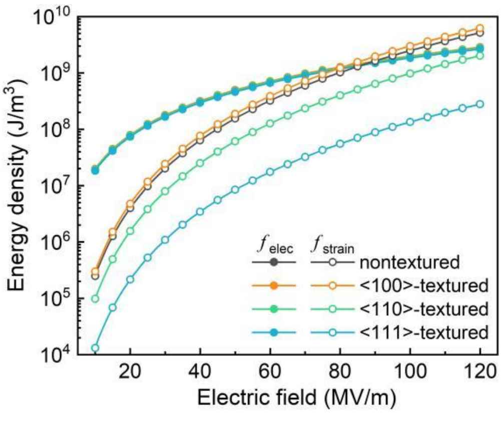

Figure S3. Comparisons of the maximum local electric energy density and the strain energy density of four samples at different applied electric fields.

4. Effects of Q_{ijkl} on the stress field and corresponding strain energy density distributions

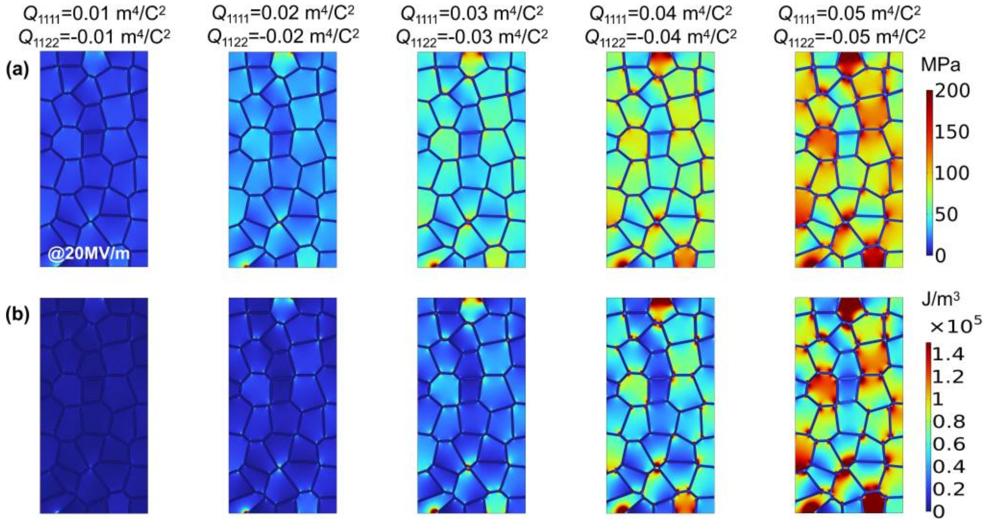

Figure S4. Local distributions of a) stress field and b) strain energy of different Q_{ijkl} under 20 MV m^{-1}.

5. Electrical and mechanical responses under different grain size (*G*)

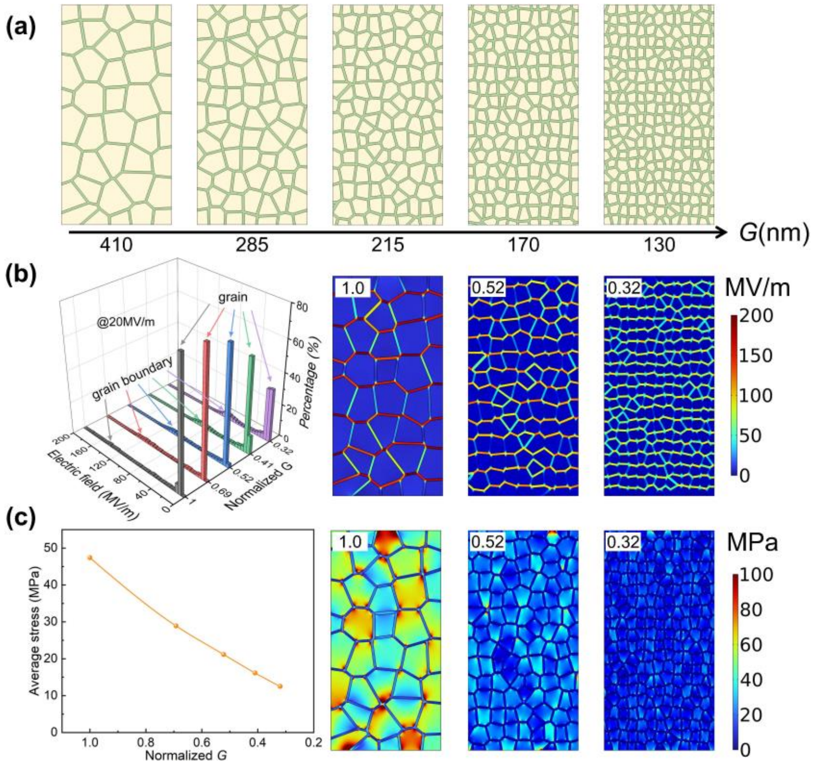

Figure S5. a) Schematic illustrations of ceramics with different *G*. b) The statistical distributions of local electric field in <100>-textured sample with different normalized *G* under 20 MV m^{-1} and local electric field distributions with normalized *G* of 1.0, 0.52 and 0.32. c) Average stress in <100>-textured sample with different normalized *G* under 20 MV m^{-1} and local stress field distributions with normalized *G* of 1.0, 0.52 and 0.32.

6. Electrical and mechanical responses under different grain boundary volume fraction

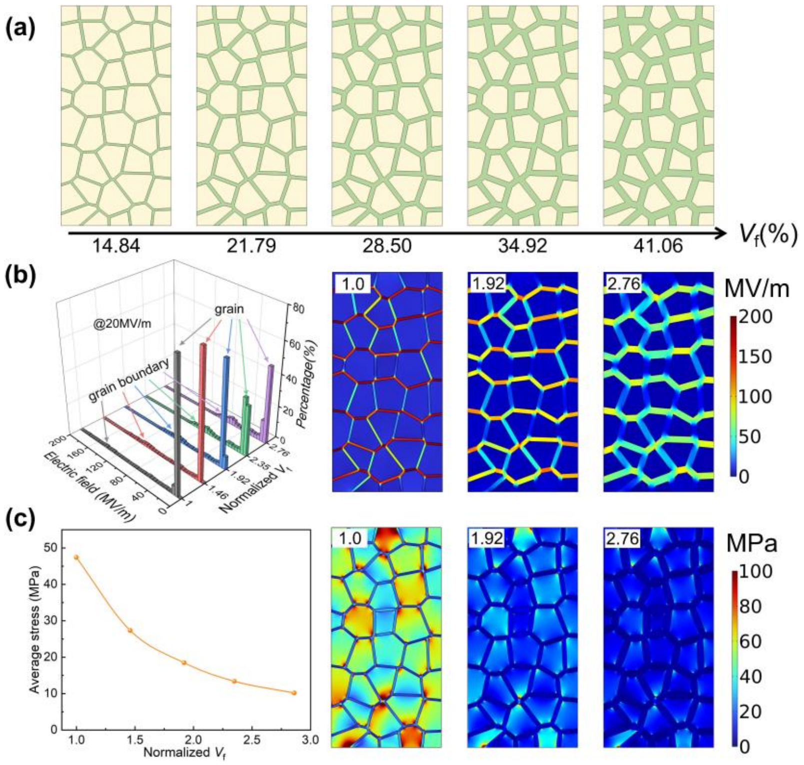

Figure S6. a) Schematic illustrations of ceramics with different V_f. b) The statistical distributions of local electric field in <100>-textured sample with different normalized V_f under 20 MV m^{-1} and local electric field distributions with normalized V_f of 1.0, 1.92 and 2.76. c) Average stress in <100>-textured sample with different normalized V_f under 20 MV m^{-1} and local stress field distributions with normalized V_f of 1.0, 1.92 and 2.76.

7. Electrical and mechanical responses under different grain shape

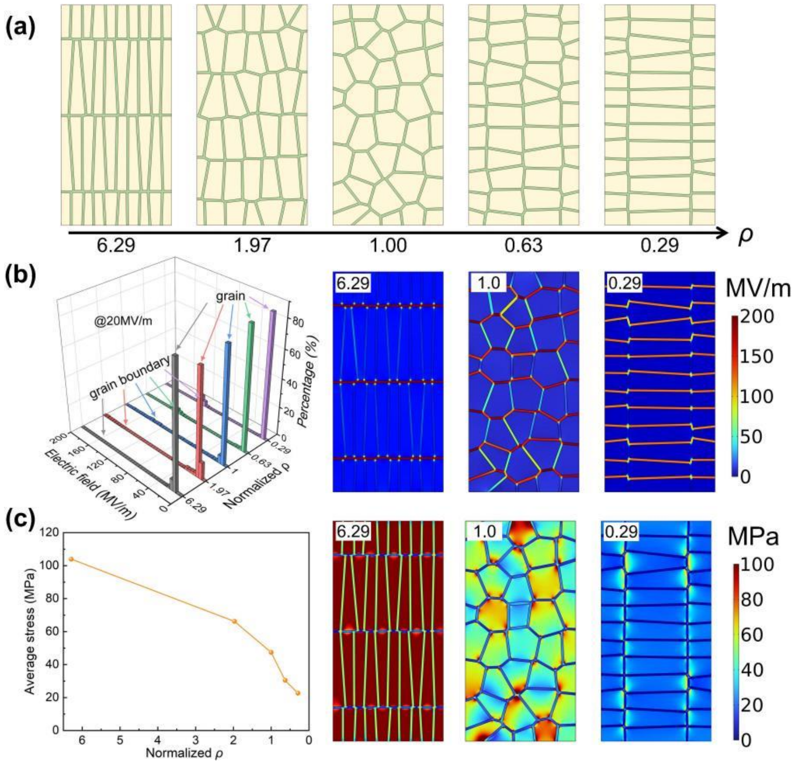

Figure S7. a) Schematic illustrations of ceramics with different ρ. b) The statistical distributions of local electric field in <100>-textured sample with different normalized ρ under 20 MV m^{-1} and local electric field distributions with normalized ρ of 6.29, 1.0 and 0.29. c) Average stress in <100>-textured sample with different normalized ρ under 20 MV m^{-1} and local stress field distributions with normalized ρ of 6.29, 1.0 and 0.29.

8. Microstructure effects of grain size, grain boundary and grain shape on the breakdown strength of textured ceramics.

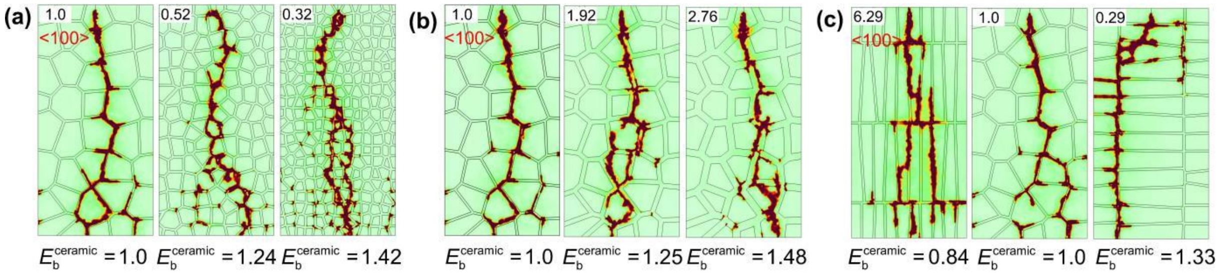

Figure S8. The evolution of electrical trees in <100>-textured sample with a) normalized G of 1.0, 0.52 and 0.32, b) normalized V_f of 1.0, 1.92 and 2.76, and c) normalized ρ of 6.29, 1.0 and 0.29.

9. The positive correlation between Q_{1111} and Q_{1122}

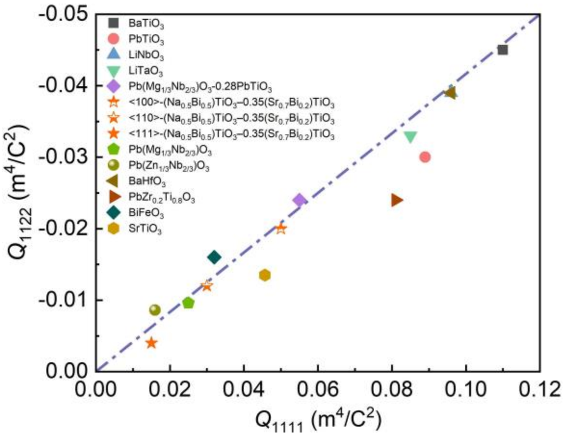

Figure S9. Comparisons of Q_{1111} and Q_{1122} of different ceramics. The correspondences of the references in the figure are as follows: $BaTiO_3$,[1] $PbTiO_3$,[2] $LiNbO_3$ and $LiTaO_3$,[3] $BaHfO_3$,[4] $PbZr_{0.2}Ti_{0.8}O_3$,[5] $BiFeO_3$,[6] $SrTiO_3$,[7] $Pb(Mg_{1/3}Nb_{2/3})O_3$-0.28$PbTiO_3$,[8] $Pb(Mg_{1/3}Nb_{2/3})O_3$,[9] $Pb(Zn_{1/3}Nb_{2/3})O_3$,[10] and $Na_{0.5}Bi_{0.5}TiO_3$-0.35$Sr_{0.7}Bi_{0.2}TiO_3$.[11]

10. Regression Analysis

To begin with, the 1st screening is conducted to find the best combination of each variable with one function by ranking the coefficients of determination R^2 by LSR, and the regression results are shown in **Table S1**. It is found that all the regression results of each variable combined with only one prototypical function show low R^2, of which $x^{1/2}$, x, $x^{-1/2}$, x^2 and ln(x) functions can lead to a R^2 of 0.115, 0.113, 0.197, 0.283 and 0.233 for Q_{1111}, Q_{1122}, G, V_f and ρ, indicating that $E_b^{creamic}$ can not be expressed by only one variable and dielectric breakdown process is affected by many factors. Based on the 1st screening, we choose top three functions of each variable to form the composite descriptors with two dependent variables in 2nd regression and screening, the regression results are shown in **Table S2**. It can been seen that R^2 has a significant improvement when considering two variables, where G with $x^{-1/2}$ function and V_f with x^2 function can result in a better R^2 = 0.658. Therefore, it can be expected that the increase in the number of independent variables could cause a higher R^2. Furthermore, to find the best predictive expression, three, four and five variables with top three functions are carried out, respectively. After five rounds of regression and screening, Q_{1111} & Q_{1122} & G & V_f & ρ with orderly $x^{1/2}$ & x & $x^{-1/2}$ & x^2 & ln(x) exhibit the highest R^2=0.950, as shown in **Table S5**. Then, based on the results of 5th round of regressions, the interactions among variables with corresponding best prototypical functions are considered, and the results are shown in **Table S6**. When all interactions are taken into account, the best R^2 = 0.953 can be achieved. Since R^2 does not changes significantly after considering the

interactions, we finally choose a simple and practical expression without considering the interaction among variables.

Table S1. The coefficient of determination R^2 in 1st round of least square regressions with only one variable and one of the 12 prototypical functions.

Descriptor	R^2(only Q_{1111})	R^2(only Q_{1122})	R^2(only G)	R^2(only V_f)	R^2(only ρ)
x	0.112	0.113	0.182	0.268	0.227
x^{-1}	0.105	0.096	0.199	0.223	0.112
$x^{1/2}$	0.115	0.112	0.188	0.258	0.244
$x^{-1/2}$	0.110	0.102	0.197	0.235	0.175
x^2	0.108	0.108	0.170	0.283	0.193
x^{-2}	0.097	0.088	0.197	0.202	0.054
x^3	0.097	0.096	0.160	0.288	0.178
x^{-3}	0.090	0.084	0.187	0.185	0.041
$\ln(x)$	0.113	0.108	0.193	0.247	0.233
$\ln(x)^{-1}$	/	/	/	/	/
e^x	0.111	0.111	0.175	0.287	0.172
e^{-x}	0.115	0.112	0.190	0.232	0.210

Table S2. The coefficient of determination R^2 in 2nd round of least square regressions with two fingerprint and corresponding three best prototypical functions selected from **Table S1**

Descriptor	R^2(Q_{1111}&G)	Descriptor	R^2(Q_{1111}&V_f)	Descriptor	R2(Q_{1111}&ρ)
$x^{1/2}$& x^{-1}	0.314	$x^{1/2}$&x^2	0.398	$x^{1/2}$&x	0.342
$x^{1/2}$&$x^{-1/2}$	0.312	$x^{1/2}$&x^3	0.403	$x^{1/2}$&$x^{1/2}$	0.359
$x^{1/2}$&x^{-2}	0.311	$x^{1/2}$&e^x	0.401	$x^{1/2}$&ln(x)	0.347
ln(x)&x^{-1}	0.312	ln(x)&x^2	0.396	ln(x)&x	0.340
ln(x)&$x^{-1/2}$	0.310	ln(x)&x^3	0.402	ln(x)&$x^{1/2}$	0.358
ln(x)&x^{-2}	0.309	ln(x)&e^x	0.400	ln(x)&ln(x)	0.346
e^{-x}&x^{-1}	0.314	e^{-x}&x^2	0.398	e^{-x}&x	0.342
e^{-x}&$x^{-1/2}$	0.312	e^{-x}&x^3	0.403	e^{-x}&$x^{1/2}$	0.359
e^{-x}&x^{-2}	0.311	e^{-x}&e^x	0.401	e^{-x}&ln(x)	0.347
Descriptor	R^2(Q_{1122}&G)	Descriptor	R^2(Q_{1122}&V_f)	Descriptor	R2(Q_{1122}&ρ)
x&x^{-1}	0.313	x&x^2	0.397	x&x	0.341
x&$x^{-1/2}$	0.311	x&x^3	0.402	x&$x^{1/2}$	0.358
x&x^{-2}	0.310	x&e^x	0.400	x&ln(x)	0.346
$x^{1/2}$&x^{-1}	0.311	$x^{1/2}$&x^2	0.395	$x^{1/2}$&x	0.339
$x^{1/2}$&$x^{-1/2}$	0.309	$x^{1/2}$&x^3	0.400	$x^{1/2}$&$x^{1/2}$	0.356
$x^{1/2}$&x^{-2}	0.308	$x^{1/2}$&e^x	0.398	$x^{1/2}$&ln(x)	0.344
e^{-x}&x^{-1}	0.311	e^{-x}&x^2	0.395	e^{-x}&x	0.339
e^{-x}&$x^{-1/2}$	0.309	e^{-x}&x^3	0.400	e^{-x}&$x^{1/2}$	0.356
e^{-x}&x^{-2}	0.308	e^{-x}&e^x	0.398	e^{-x}&ln(x)	0.344
Descriptor	R^2(G&V_f)	Descriptor	R^2(V_f&ρ)	Descriptor	R^2(ρ&G)
x^{-1}&x^2	0.652	x^2&x	0.452	x&x^{-1}	0.377
x^{-1}&x^3	0.645	x^2&$x^{1/2}$	0.479	x&$x^{-1/2}$	0.374
x^{-1}&e^x	0.644	x^2&ln(x)	0.493	x&x^{-2}	0.378
$x^{-1/2}$&x^2	0.658	x^3&x	0.460	$x^{1/2}$&x^{-1}	0.402
$x^{-1/2}$&x^3	0.649	x^3&$x^{1/2}$	0.487	$x^{1/2}$&$x^{-1/2}$	0.399
$x^{-1/2}$&e^x	0.649	x^3&ln(x)	0.500	$x^{1/2}$&x^{-2}	0.403
x^{-2}&x^2	0.630	e^x&x	0.458	lnx&x^{-1}	0.412
x^{-2}&x^3	0.625	e^x&$x^{1/2}$	0.485	lnx&$x^{-1/2}$	0.410
x^{-2}&e^x	0.625	e^x&ln(x)	0.498	lnx&x^{-2}	0.412

Table S3. The coefficient of determination R^2 (R^2>0.7) in 3rd round of least square regressions with three fingerprint and corresponding three best prototypical functions selected from **Table S1**

Descriptor	$R^2(Q_{1111}\&G\&V_f)$	Descriptor	$R^2(Q_{1111}\&G\&V_f)$	Descriptor	$R^2(Q_{1111}\&G\&V_f)$
$x^{1/2}\&x^{-1}\&x^{2}$	0.767	$\ln(x)\&x^{-1}\&x^{2}$	0.765	$e^{-x}\&x^{-1}\&x^{2}$	0.767
$x^{1/2}\&x^{-1}\&x^{3}$	0.759	$\ln(x)\&x^{-1}\&x^{3}$	0.758	$e^{-x}\&x^{-1}\&x^{3}$	0.759
$x^{1/2}\&x^{-1}\&e^{x}$	0.759	$\ln(x)\&x^{-1}\&e^{x}$	0.758	$e^{-x}\&x^{-1}\&e^{x}$	0.759
$x^{1/2}\&x^{-1/2}\&x^{2}$	0.773	$\ln(x)\&x^{-1/2}\&x^{2}$	0.771	$e^{-x}\&x^{-1/2}\&x^{2}$	0.773
$x^{1/2}\&x^{-1/2}\&x^{3}$	0.764	$\ln(x)\&x^{-1/2}\&x^{3}$	0.762	$e^{-x}\&x^{-1/2}\&x^{3}$	0.764
$x^{1/2}\&x^{-1/2}\&e^{x}$	0.764	$\ln(x)\&x^{-1/2}\&e^{x}$	0.762	$e^{-x}\&x^{-1/2}\&e^{x}$	0.764
$x^{1/2}\&x^{-2}\&x^{2}$	0.746	$\ln(x)\&x^{-2}\&x^{2}$	0.744	$e^{-x}\&x^{-2}\&x^{2}$	0.746
$x^{1/2}\&x^{-2}\&x^{3}$	0.740	$\ln(x)\&x^{-2}\&x^{3}$	0.738	$e^{-x}\&x^{-2}\&x^{3}$	0.740
$x^{1/2}\&x^{-2}\&e^{x}$	0.739	$\ln(x)\&x^{-2}\&e^{x}$	0.738	$e^{-x}\&x^{-2}\&e^{x}$	0.739
Descriptor	$R^2(Q_{1122}\&G\&V_f)$	Descriptor	$R^2(Q_{1122}\&G\&V_f)$	Descriptor	$R^2(Q_{1122}\&G\&V_f)$
$x\&x^{-1}\&x^{2}$	0.766	$x^{1/2}\&x^{-1}\&x^{2}$	0.764	$e^{-x}\&x^{-1}\&x^{2}$	0.764
$x\&x^{-1}\&x^{3}$	0.758	$x^{1/2}\&x^{-1}\&x^{3}$	0.757	$e^{-x}\&x^{-1}\&x^{3}$	0.756
$x\&x^{-1}\&e^{x}$	0.758	$x^{1/2}\&x^{-1}\&e^{x}$	0.756	$e^{-x}\&x^{-1}\&e^{x}$	0.756
$x\&x^{-1/2}\&x^{2}$	0.771	$x^{1/2}\&x^{-1/2}\&x^{2}$	0.770	$e^{-x}\&x^{-1/2}\&x^{2}$	0.770
$x\&x^{-1/2}\&x^{3}$	0.763	$x^{1/2}\&x^{-1/2}\&x^{3}$	0.761	$e^{-x}\&x^{-1/2}\&x^{3}$	0.761
$x\&x^{-1/2}\&e^{x}$	0.763	$x^{1/2}\&x^{-1/2}\&e^{x}$	0.761	$e^{-x}\&x^{-1/2}\&e^{x}$	0.761
$x\&x^{-2}\&x^{2}$	0.745	$x^{1/2}\&x^{-2}\&x^{2}$	0.743	$e^{-x}\&x^{-2}\&x^{2}$	0.743
$x\&x^{-2}\&x^{3}$	0.739	$x^{1/2}\&x^{-2}\&x^{3}$	0.737	$e^{-x}\&x^{-2}\&x^{3}$	0.737
$x\&x^{-2}\&e^{x}$	0.738	$x^{1/2}\&x^{-2}\&e^{x}$	0.737	$e^{-x}\&x^{-2}\&e^{x}$	0.736
Descriptor	$R^2(G\&V_f\&\rho)$	Descriptor	$R^2(G\&V_f\&\rho)$	Descriptor	$R^2(G\&V_f\&\rho)$
$x^{-1}\&x^{2}\&x$	0.749	$x^{-1}\&x^{2}\&x^{1/2}$	0.752	$x^{-1}\&x^{2}\&\ln(x)$	0.735
$x^{-1}\&x^{3}\&x$	0.758	$x^{-1}\&x^{3}\&x^{1/2}$	0.787	$x^{-1}\&x^{3}\&\ln(x)$	0.770
$x^{-1}\&e^{x}\&x$	0.831	$x^{-1}\&e^{x}\&x^{1/2}$	0.835	$x^{-1}\&e^{x}\&\ln(x)$	0.813
$x^{-1/2}\&x^{2}\&x$	0.747	$x^{-1/2}\&x^{2}\&x^{1/2}$	0.749	$x^{-1/2}\&x^{2}\&\ln(x)$	0.734
$x^{-1/2}\&x^{3}\&x$	0.782	$x^{-1/2}\&x^{3}\&x^{1/2}$	0.784	$x^{-1/2}\&x^{3}\&\ln(x)$	0.768
$x^{-1/2}\&e^{x}\&x$	0.826	$x^{-1/2}\&e^{x}\&x^{1/2}$	0.829	$x^{-1/2}\&e^{x}\&\ln(x)$	0.809
$x^{-2}\&x^{2}\&x$	0.746	$x^{-2}\&x^{2}\&x^{1/2}$	0.748	$x^{-2}\&x^{2}\&\ln(x)$	0.733
$x^{-2}\&x^{3}\&x$	0.781	$x^{-2}\&x^{3}\&x^{1/2}$	0.783	$x^{-2}\&x^{3}\&\ln(x)$	0.767
$x^{-2}\&e^{x}\&x$	0.825	$x^{-2}\&e^{x}\&x^{1/2}$	0.829	$x^{-2}\&e^{x}\&x^{1/2}$	0.808

Table S4. The coefficient of determination R^2 (R^2>0.9) in 4th round of least square regressions with four fingerprint and corresponding three best prototypical functions selected from **Table S1**

Descriptor	$R^2(Q_{1111}\&G\&V_f\&\rho)$	Descriptor	$R^2(Q_{1111}\&G\&V_f\&\rho)$
$x^{1/2}\&x^{-1}\&x^{2}\&\ln(x)$	0.945	$\ln(x)\&x^{-1/2}\&e^{x}\&\ln(x)$	0.942
$x^{1/2}\&x^{-1}\&x^{3}\&\ln(x)$	0.940	$\ln(x)\&x^{-2}\&x^{2}\&\ln(x)$	0.926
$x^{1/2}\&x^{-1}\&e^{x}\&\ln(x)$	0.940	$\ln(x)\&x^{-2}\&x^{3}\&\ln(x)$	0.922
$x^{1/2}\&x^{-1/2}\&x^{3}\&\ln(x)$	0.944	$e^{-x}\&x^{-1}\&x^{2}\&\ln(x)$	0.945
$x^{1/2}\&x^{-1/2}\&e^{x}\&\ln(x)$	0.943	$e^{-x}\&x^{-1}\&x^{3}\&\ln(x)$	0.940
$x^{1/2}\&x^{-2}\&x^{2}\&\ln(x)$	0.928	$e^{-x}\&x^{-1}\&e^{x}\&\ln(x)$	0.940
$x^{1/2}\&x^{-2}\&x^{3}\&\ln(x)$	0.924	$e^{-x}\&x^{-1/2}\&x^{2}\&\ln(x)$	0.948
$x^{1/2}\&x^{-2}\&e^{x}\&\ln(x)$	0.923	$e^{-x}\&x^{-1/2}\&x^{3}\&\ln(x)$	0.944
$\ln(x)\&x^{-1}\&x^{2}\&\ln(x)$	0.944	$e^{-x}\&x^{-1/2}\&e^{x}\&\ln(x)$	0.943
$\ln(x)\&x^{-1}\&x^{3}\&\ln(x)$	0.939	$e^{-x}\&x^{-2}\&x^{2}\&\ln(x)$	0.927
$\ln(x)\&x^{-1}\&e^{x}\&\ln(x)$	0.938	$e^{-x}\&x^{-2}\&x^{3}\&\ln(x)$	0.924
$\ln(x)\&x^{-1/2}\&x^{2}\&\ln(x)$	0.948	$e^{-x}\&x^{-2}\&e^{x}\&\ln(x)$	0.923
$\ln(x)\&x^{-1/2}\&x^{3}\&\ln(x)$	0.942		
Descriptor	$R^2(Q_{1122}\&G\&V_f\&\rho)$	Descriptor	$R^2(Q_{1122}\&G\&V_f\&\rho)$

$x\&x^{-1}\&x^{2}\&\ln(x)$	0.944	$x^{1/2}\&x^{-1/2}\&e^{x}\&\ln(x)$	0.941
$x\&x^{-1}\&x^{3}\&\ln(x)$	0.939	$x^{1/2}\&x^{-2}\&x^{2}\&\ln(x)$	0.925
$x\&x^{-1}\&e^{x}\&\ln(x)$	0.930	$x^{1/2}\&x^{-2}\&x^{3}\&\ln(x)$	0.921
$x\&x^{-1/2}\&x^{3}\&\ln(x)$	0.943	$e^{-x}\&x^{-1}\&x^{2}\&\ln(x)$	0.942
$x\&x^{-1/2}\&e^{x}\&\ln(x)$	0.942	$e^{-x}\&x^{-1}\&x^{3}\&\ln(x)$	0.937
$x\&x^{-2}\&x^{2}\&\ln(x)$	0.926	$e^{-x}\&x^{-1}\&e^{x}\&\ln(x)$	0.937
$x\&x^{-2}\&x^{3}\&\ln(x)$	0.923	$e^{-x}\&x^{-1/2}\&x^{2}\&\ln(x)$	0.946
$x\&x^{-2}\&e^{x}\&\ln(x)$	0.922	$e^{-x}\&x^{-1/2}\&x^{3}\&\ln(x)$	0.941
$x^{1/2}\&x^{-1}\&x^{2}\&\ln(x)$	0.943	$e^{-x}\&x^{-1/2}\&e^{x}\&\ln(x)$	0.940
$x^{1/2}\&x^{-1}\&x^{3}\&\ln(x)$	0.938	$e^{-x}\&x^{-2}\&x^{2}\&\ln(x)$	0.925
$x^{1/2}\&x^{-1}\&e^{x}\&\ln(x)$	0.937	$e^{-x}\&x^{-2}\&x^{3}\&\ln(x)$	0.921
$x^{1/2}\&x^{-1/2}\&x^{2}\&\ln(x)$	0.947	$e^{-x}\&x^{-2}\&e^{x}\&\ln(x)$	0.920
$x^{1/2}\&x^{-1/2}\&x^{3}\&\ln(x)$	0.941		

Table S5. The coefficient of determination R^2 (R^2>0.940) in 5th round of least square regressions with five fingerprint and corresponding three best prototypical functions selected from **Table S1**

Descriptor	$R^2(Q_{1111}\&Q_{1122}\&G\&V_f\&\rho)$	Descriptor	$R^2(Q_{1111}\&Q_{1122}\&G\&V_f\&\rho)$
$x^{1/2}\&x\&x^{-1}\&x^{2}\&\ln(x)$	0.944	$x^{1/2}\&e^{-x}\&x^{-1/2}\&e^{x}\&\ln(x)$	0.942
$x^{1/2}\&x\&x^{-1/2}\&x^{2}\&\ln(x)$	0.950	$\ln(x)\&x\&x^{-1}\&x^{2}\&\ln(x)$	0.944
$x^{1/2}\&x\&x^{-1/2}\&x^{3}\&\ln(x)$	0.943	$\ln(x)\&x\&x^{-1/2}\&x^{2}\&\ln(x)$	0.948
$x^{1/2}\&x\&x^{-1/2}\&e^{x}\&\ln(x)$	0.942	$\ln(x)\&x\&x^{-1}\&x^{3}\&\ln(x)$	0.943
$x^{1/2}\&x^{1/2}\&x^{-1}\&x^{2}\&\ln(x)$	0.944	$\ln(x)\&x\&x^{-1}\&e^{x}\&\ln(x)$	0.942
$x^{1/2}\&x^{1/2}\&x^{-1/2}\&x^{2}\&\ln(x)$	0.948	$\ln(x)\&x^{1/2}\&x^{-1}\&x^{2}\&\ln(x)$	0.943
$x^{1/2}\&x^{1/2}\&x^{-1/2}\&x^{3}\&\ln(x)$	0.942	$\ln(x)\&x^{1/2}\&x^{1/2}\&x^{2}\&\ln(x)$	0.947
$x^{1/2}\&x^{1/2}\&x^{-1/2}\&e^{x}\&\ln(x)$	0.942	$e^{-x}\&x\&x^{-1/2}\&x^{2}\&\ln(x)$	0.948
$x^{1/2}\&e^{-x}\&x^{-1}\&x^{2}\&\ln(x)$	0.944	$e^{-x}\&x\&x^{-1/2}\&x^{3}\&\ln(x)$	0.943
$x^{1/2}\&e^{-x}\&x^{-1/2}\&x^{2}\&\ln(x)$	0.948	$e^{-x}\&x^{1/2}\&x^{-1}\&x^{2}\&\ln(x)$	0.943
$x^{1/2}\&e^{-x}\&x^{-1/2}\&x^{3}\&\ln(x)$	0.943	$e^{-x}\&x^{1/2}\&x^{-1/2}\&x^{2}\&\ln(x)$	0.947

Table S6. The coefficient of determination R^2 in the 6th round least square regressions with consideration of interactions among descriptors. The total analytical function i

$$E_b^{ceramic} = \beta_0 + \beta_1 Q_{1111}^{1/2} + \beta_2 Q_{1122} + \beta_3 G^{-1/2} + \beta_4 V_f^{\ 2} + \beta_5 \ln\rho + \beta_6 Q_{1111} Q_{1122} + \beta_7 G^{-1/2} V_f^{\ 2} + \beta_8 G^{-1/2} \ln\rho$$
$$+ \beta_9 V_f^{\ 2} \ln\rho + \beta_{10} Q_{1111}^{1/2} Q_{1122} G^{-1/2} + \beta_{11} Q_{1111}^{1/2} Q_{1122} V_f^{\ 2} + \beta_{12} Q_{1111}^{1/2} Q_{1122} \ln\rho + \beta_{13} Q_{1111}^{1/2} Q_{1122} G^{-1/2} V_f^{\ 2} \ln\rho$$

	None	$Q_{1111}\&Q_{1122}$	$G\&V_f$	$G\&\rho$	$V_f\&\rho$	$Q_{1111}\&Q_{1122}\&G$	$Q_{1111}\&Q_{1122}\&V_f$	$Q_{1111}\&Q_{1122}\&\rho$	$Q_{1111}\&Q_{1122}\&G\&V_f\&\rho$	All
R^2	0.950	0.951	0.950	0.950	0.950	0.952	0.950	0.951	0.951	0.953
B_0	1.179	1.326	1.371	0.179	1.179	1.230	1.179	1.180	1.180	1.468
β_1	-1.098	-1.486	-1.098	-1.098	-1.098	-1.408	-1.098	-1.101	-1.101	-1.468
β_2	0.392	0.781	0.392	0.392	0.392	0.703	0.392	0.394	0.394	0.781
β_3	0.508	0.508	0.316	0.508	0.508	0.566	0.508	0.508	0.508	0.355
β_4	0.068	0.068	-0.124	0.068	0.068	0.068	0.068	0.068	0.068	-0.142
β_5	-0.138	-0.138	-0.138	-0.069	-0.069	-0.138	-0.138	-0.128	-0.128	-0.042
β_6	/	-0.153	/	/	/	/	/	/	/	-0.023
β_7	/	/	0.192	/	/	/	/	/	/	0.212
β_8	/	/	/	-0.069	/	/	/	/	/	-0.042
β_9	/	/	/	/	-0.069	/	/	/	/	-0.042
β_{10}	/	/	/	/	/	-0.108	/	/	/	-0.109
β_{11}	/	/	/	/	/	/	0.000	/	/	-0.003
β_{12}	/	/	/	/	/	/	/	-0.018	/	-0.010

β_{13}	/	/	/	/	/	/	/	/	-0.018	-0.010

[1] J. Wang, F. Meng, X. Ma, M. Xu, L. Chen, *J. Appl. Phys.* **2010**, 108, 034107.

[2] M. J. Haun, E. Furman, S. Jang, H. McKinstry, L. Cross, *J. Appl. Phys.* **1987**, 62, 3331.

[3] T. Yamada, *J. Appl. Phys.* **1972**, 43, 328.

[4] C.-H. Ma, Y.-K. Liao, Y. Zheng, S. Zhuang, S.-C. Lu, P.-W. Shao, J.-W. Chen, Y.-H. Lai, P. Yu, J.-M. Hu, H. Rong, Y.-H. Chu, *ACS Appl. Mater. Interfaces* **2022**, 14, 22278.

[5] Y. Li, S. Choudhury, Z. Liu, L. Chen, *Appl. Phys. Lett.* **2003**, 83, 1608.

[6] L. Li, X. Cheng, J. R. Jokisaari, P. Gao, J. Britson, C. Adamo, C. Heikes, D. G. Schlom, L.-Q. Chen, X. Pan, *Phys. Rev. Lett.* **2018**, 120, 137602.

[7] G. Sheng, Y. Li, J. Zhang, S. Choudhury, Q. Jia, V. Gopalan, D. G. Schlom, Z. Liu, L. Chen, *J. Appl. Phys.* **2010**, 108, 084113.

[8] F. Li, L. Jin, Z. Xu, D. Wang, S. Zhang, *Appl. Phys. Lett.* **2013**, 102, 152910.

[9] S.-G. Lee, R. G. Monteiro, R. S. Feigelson, H. S. Lee, M. Lee, S.-E. Park, *Appl. Phys. Lett.* **1999**, 74, 1030.

[10] S. Nomura, J. Kuwata, S. Jang, L. Cross, R. Newnham, *Mater. Res. Bull.* **1979**, 14, 769.

[11] J. Li, Z. Shen, X. Chen, S. Yang, W. Zhou, M. Wang, L. Wang, Q. Kou, Y. Liu, Q. Li, Z. Xu, C. Yunfei, S. Zhang, F. Li, *Nat. Mater.* **2020**, 19, 999.
